# Radiographical characteristics and traction duration of impacted maxillary canine requiring surgical exposure and orthodontic traction: a cross-sectional study

**DOI:** 10.1038/s41598-022-23232-7

**Published:** 2022-11-10

**Authors:** Jin-Seob Yang, Jung-Yul Cha, Ji-Yeon Lee, Sung-Hwan Choi

**Affiliations:** 1 Department of Orthodontics, National Health Insurance Service Ilsan Hospital, Goyang, 10444 Republic of Korea; 2 Department of Orthodontics, Institute of Craniofacial Deformity, Yonsei University College of Dentistry, Seoul, 03722 Republic of Korea

**Keywords:** Malocclusion, Oral pathology

## Abstract

This cross-sectional study aimed to classify the radiographical characteristics of impacted maxillary canines that were surgically exposed following orthodontic traction and to find out which factor is most closely related to traction duration. This study enrolled 74 patients with 87 maxillary canines. Cone-beam computed tomography images, panoramic radiographs, and medical records were analyzed. Cystic-appearing lesion and resorption of adjacent roots were observed in 26.4% and 23.0% of cases, respectively. Impacted maxillary canines were mostly distributed in the lateral incisor area. The mean (± standard deviation) traction duration for the 47 teeth that met the study criteria was 13.9 (± 8.9) months. Impacted maxillary canines treated with surgical exposure and orthodontic traction showed increasing possibilities of palatal impaction and resorption of the adjacent root as they were located mesially (*p* < 0.05). The distance from the occlusal plane to the impacted maxillary canine showed the strongest positive correlation with traction duration (*r* = 0.519, *p* < 0.01). When establishing treatment plans for patients with impacted maxillary canines, distance from the occlusal plane to the impacted canines, rather than the angle, should be considered in predicting the duration of treatment.

## Introduction

The maxillary canine is an important tooth that determines the shape, function, and occlusal structure of the dental arch^1^. Maxillary canine impaction occurs frequently because of the long eruption pathway that the tooth has to follow: from the site of the tooth bud to the final eruption site^2^. A previous study reported that the prevalence of impacted maxillary canines varies by region (0.92–2.1%)^3–5^. Impacted maxillary canines are observed more in women than in men, and bilateral impaction is observed in approximately 17–45% of all individuals with impacted maxillary canines^6^. The proportions of buccal and palatal impactions were high in Eastern and Western people, respectively^7^. If left untreated, impacted maxillary canines can lead to a variety of complications, such as resorption of the adjacent root, displacement, and cystic-appearing lesion formation^8,9^. Therefore, early diagnosis and proper intervention are necessary to prevent these complications. Moreover, the need for the treatment of impacted maxillary canines is highest in the Index of Orthodontic Treatment Need classification, which classifies the need for orthodontic treatment^10^. Therefore, proper intervention by orthodontists is necessary. Orthodontists must have an understanding of various two- or three-dimensional aspects related to the positioning of the impacted tooth within the dental arch to treat impacted maxillary canines; it is also necessary to have a rough understanding of the difficulty level of traction using radiographs to ensure successful orthodontic traction.

Several previous reports have reported on the appropriate timing for early intervention in impacted maxillary canines. These studies have mostly focused on the prediction of whether unerupted canines can erupt normally using panoramic radiographs^11–13^ or the evaluation of the effectiveness of panoramic radiographs by comparing its results with that of cone-beam computed tomography (CBCT) in the analysis of various aspects of maxillary canine impaction^14,15^. The participants of these studies had developed unerupted canines that later erupted normally; thus, there is a limitation that the common characteristics of the patients were obscured. Furthermore, the correlation between the characteristics of impacted maxillary canines that require orthodontic intervention and traction duration remains unclear.

This cross-sectional study aimed to classify the radiographical characteristics of impacted maxillary canines diagnosed as “impacted canines” that were subjected to surgical exposure and orthodontic traction. In addition, to provide advice for successful traction of impacted maxillary canines, this study investigated the correlation between these characteristics and the traction duration required for impacted maxillary canines to find out which factor, distance or angle of the impacted canines, is most closely related with traction duration. The null hypothesis of this study was that the orthodontic traction duration of impacted maxillary canines is not correlated to their radiographical characteristics.

## Results

### Descriptive statistics for subjects

In total, 74 patients and 87 impacted maxillary canines were included in this study (Table 1).

**Table 1:** Descriptive statistics for study population.

Variables		N	%
Age (year)†	< 12	48	64.9
	≥ 12	26	35.1
	Total	74	100
Gender†	Male	32	43.2
	Female	42	56.8
	Total	74	100
Skeletal Class†	Class I (ANB 0°–4°)	27	36.5
	Class II (ANB > 4°)	23	31.1
	Class III (ANB < 0°)	24	32.4
	Total	74	100
Type of impaction†	Unilateral	61	82.4
	Bilateral	13	17.6
	Total	74	100
Impaction site (Right or Left)‡	Right	45	51.7
	Left	42	48.3
	Total	87	100

^†^Total number of patients (n = 74).

^‡^Total number of teeth = 87.

The mean (± standard deviation [SD]) age at which surgical exposure was performed was 10.9 (± 2.4) years. Forty-eight (64.9%) patients who underwent surgical exposure were aged < 12 years. This study included 32 male (43.2%) and 42 female (56.8%) patients. The distributions according to skeletal classification for Classes I, III, and II were 27 (36.5%), 24 (32.4%), and 23 (31.1%), respectively. In total, 61 (82.4%) and 13 (17.6%) patients had unilateral and bilateral impactions, respectively. Moreover, 45 (51.7%) and 42 (48.3%) patients had right- and left-side impactions, respectively.

### CBCT findings associated with impacted maxillary canines

CBCT findings associated with impacted maxillary canines were also investigated (Table 2).

**Table 2:** CBCT findings associated with impacted maxillary canines.

Variables		N	%
Impaction site	Buccal	65	74.7
	Palatal	22	25.3
Cystic-appearing lesion		23	26.4
Resorption of adjacent root		20	23.0
Supernumerary teeth		4	4.6
Odontoma		4	4.6

Total number of teeth = 87.

In the bucco-palatal position, 65 (74.7%) and 22 (25.3%) canines were buccally and palatally impacted, respectively. Cystic-appearing lesion was associated with 23 teeth (26.4%), followed by root resorption of the adjacent teeth in 20 (23.0%), and either odontoma or supernumerary teeth with 4 (4.6%).

The distribution of impacted maxillary canines according to the sector was also classified (Table 3). Impacted maxillary canines were distributed in sector B, the lateral incisor area, with the highest number being 53 (60.9%). This was followed by 18 (20.7%), 9 (10.3%), and 7 (8.1%) canines in sectors A, D, and C, respectively.

**Table 3:** Classification of impacted maxillary canines by sectors.

	N	%
Sector A	18	20.7
Sector B	53	60.9
Sector C	7	8.1
Sector D	9	10.3
Total	87	100

The ratio of CBCT findings associated with impacted maxillary canines by sector was analyzed (Table 4).

**Table 4:** Distribution of CBCT findings associated with impacted maxillary canines within sectors.

		Sector A	Sector B	Sector C	Sector D	Total	*p*-value
Impaction site	Buccal	8 (12.3%)	44 (67.7%)	4 (6.2%)	9 (13.8%)	65	0.010
	Palatal	10 (45.5%)	9 (40.9%)	3 (13.6%)	0	22	
Cystic-appearing lesion		3 (13.0%)	16 (69.6%)	1 (4.3%)	3 (13.0%)	23	0.533
Root resorption of adjacent root		9 (45.0%)	9 (45.0%)	0	2 (10.0%)	20	0.045
SNT or odontoma		1 (12.5%)	4 (50.0%)	2 (25.0%)	1 (12.5%)	8	0.297

Values are presented as number (%). SNT, supernumerary teeth. *P*-values were calculated using a linear-by-linear association test.

Of the 65 buccally impacted teeth, 44 (67.7%) were distributed in sector B based on the ratio of bucco-palatal impaction. Of the 22 palatally impacted teeth, 10 (45.5%) were distributed in sector A. No palatally impacted canines were distributed in sector D. The 23 and 20 impacted maxillary canines associated with cystic-appearing lesion and root resorption of adjacent teeth had the highest number of 16 (69.6%) in sector B and 9 (45.0%) in sectors A and B, respectively. Most of the eight impacted maxillary canines associated with odontoma or supernumerary teeth were in sector B (50.0%). There was a significant correlation between the sector and the ratio of bucco-palatal impaction and root resorption of the adjacent teeth (*p* < 0.05). The ratio of palatally impacted canines and root resorption of adjacent teeth increased as they moved to sector A because the impacted maxillary canines were located mesially.

The angle between the long axis of the impacted maxillary canine and the occlusal plane (3^OP) and the midline (3^ML) plane and the distance from the cusp tip of the impacted maxillary canine to the midline (3c-ML) showed statistically significant differences for each sector (*p* < 0.001) (Table 5).

**Table 5:** Comparison of measurements by sectors in panoramic radiographs.

Measurement		Sector A	Sector B	Sector C	Sector D	*p*-value
3⌃OP (°)	Mean [± SD]	31.3 [± 14.2]^a^	49.7 [± 13.3]^b^	66.7 [± 8.8]^c^	72.4 [± 10.4]^c^	< 0.001
	Range	11.1–53.6	6.4–73.3	54.3–77.5	52.4–84.5	
3⌃ML (°)	Mean [± SD]	46.3 [± 15.8]^c^	27.0 [± 13.4]^b^	8.0 [± 6.9]^a^	15.8 [± 16.0]^b^	< 0.001
	Range	16.2–68.7	5.9–66.5	1.2–19.6	0.2–48.7	
3c-OP (mm)	Mean [± SD]	13.3 [± 3.2]	11.7 [± 3.3]	10.1 [± 2.8]	12.0 [± 3.7]	0.260
	Range	6.9–18.5	3.5–22.4	6–14	5.9–18.1	
3c-ML (mm)	Mean [± SD]	3.8 [± 2.1]^a^	9.0 [± 2.1]^b^	12.1 [± 2.2]^c^	20.4 [± 2.8]^d^	< 0.001
	Range	0–6.9	5–13.3	9.7–16.4	15.5–25.8	

*P*-values were calculated using one-way analysis of variance with Tukey’s post-hoc test. Significant differences within each row are represented by the lowercase letters. SD, standard deviation.

3^OP was the largest in sectors C and D at 66.7 (± 8.8)° and 72.4 (± 10.4)°, respectively, and the smallest at 31.3 (± 14.2)° in sector A. The greater the position of the impacted canines located distally, the greater was the angle formed with the occlusal plane. 3^ML was the largest in sector A at 46.3 (± 15.8)° and the smallest at 8.0 (± 6.9)° in sector C.

The closer the position of the impacted canine to the area of the deciduous canine, the smaller was the angle formed with the midline; the farther the position of the impacted canine to the area of the deciduous canine, the greater was the angle formed with the midline. 3c-ML was the largest in sector D at 20.4 (± 2.8) mm and the smallest in sector A at 3.8 (± 2.1) mm. The distance from the midline increased when the impacted canine was located distally.

### Traction duration according to bucco-palatal position by sectors

Traction duration according to bucco-palatal impaction for each sector was compared (Table 6).

**Table 6:** Comparison of traction duration (in months) by sectors.

		N	Mean traction duration
Total	Buccal	33	12.9 [± 7.5]
	Palatal	14	16.2 [± 11.2]
	Total	47	13.9 [± 8.9]
Sector A	Buccal	3	22.7 [± 17.9]
	Palatal	7	17.3 [± 5.9]
	Total	10	18.9 [± 11.2]
Sector B	Buccal	22	12 [± 3.7]
	Palatal	5	18.4 [± 16.0]
	Total	27	13.2 [± 8.0]
Sector C	Buccal	2	6.0 [± 1.0]
	Palatal	2	7.0 [± 4.0]
	Total	4	6.5 [± 3.0]
Sector D	Buccal	6	13.5 [± 5.5]
	Palatal	0	–
	Total	6	13.5 [± 5.5]

Values are presented as mean [± standard deviation].

Of the 74 teeth that were successfully separated from the adjacent teeth and exposed to the oral cavity, 13 were either treated or lost, and 47 were arranged on a 0.016 × 0.022-inch NiTi wire, corresponding to the traction period criteria of this study. Traction duration according to bucco-palatal impaction showed no statistically significant differences in any sector. The mean (± SD) traction duration for all 47 teeth was 13.9 (± 8.9) months. The average traction durations for buccally and palatally impacted teeth were 12.9 (± 7.5) and 16.2 (± 11.2) months, respectively. In sector A, traction duration took 18.9 (± 11.2) months for 10 teeth, with averages of 22.7 (± 17.9) and 17.3 (± 5.9) months for buccally and palatally impacted teeth, respectively. In sector B, traction duration took an average of 13.2 (± 8.0) months for 27 teeth, with averages of 12 (± 3.7) and 18.4 (± 16.0) months for buccally and palatally impacted teeth, respectively. In sector C, traction duration took an average of 6.5 (± 3.0) months for four teeth, with averages of 6.0 (± 1.0) and 7.0 (± 4.0) months for buccally and palatally impacted teeth, respectively. In sector D, traction duration took an average of 13.5 (± 5.5) months for six buccally impacted teeth.

The correlation between the linear and angular measurements and traction duration was analyzed (Table 7).

**Table 7:** Correlation between panoramic radiograph measurements and traction duration.

		3^OP (°)	3^ML (°)	3c-OP (mm)	3c-ML (mm)
Traction duration (months)	R	−0.256	0.302	0.519	−0.306
	*p*-value	0.082	0.039	< 0.001	0.036

*P-*values were calculated using Pearson correlation coefficient.

### Correlation between panoramic radiograph measurements and traction duration

The Pearson correlation coefficient was used to statistically analyze the correlation between each measurement and traction duration. There was a statistically significant correlation between the distance from the cusp tip of the impacted maxillary canine to the occlusal plane (3c-OP), 3^ML, and 3c-ML with traction duration (*p* < 0.05). The 3c-OP showed the strongest correlation with traction duration (*r* = 0.519, *p* < 0.001). The factor most associated with the traction duration of the impacted maxillary canines was its distance to the occlusal plane, rather than the angle of the impacted maxillary canines.

## Discussion

This study aimed to classify the radiographical characteristics of impacted maxillary canines treated by orthodontists and to investigate the correlation between these radiographical characteristics and the traction time required for impacted maxillary canines. The distance from the maxillary canines to the occlusal plane and to the midline, and the angle between the midline and maxillary canines showed a statistically significant correlation with traction duration. The distance from the maxillary canines to the occlusal plane showed the strongest positive correlation with traction duration. Hence, the null hypothesis of this study was rejected.

Orthodontists often encounter impacted maxillary canines in clinical practice. The treatment for impacted teeth increases the duration and difficulty of the overall orthodontic treatment^8^. Impacted maxillary canines can cause various complications in the surrounding tissues if orthodontists fail to properly intervene at an early stage^9^. Before treating impacted maxillary canines, practitioners need to understand the various aspects of impaction associated with impacted maxillary canines, and it is important to use panoramic radiographs to identify radiographic information, such as the angle and location of the impacted canines.

Ericson and Kurol argued that before the age of 10 years, the determination of impaction was significantly early because of variations in eruption pathways^11^ and that the ectopic position of impacted maxillary canines should be identified before the age of 11. If the palatally impacted maxillary canine is on the distal side of the maxillary lateral incisor’s midline, 91% of these will change their path of eruption spontaneously if the deciduous canine is extracted at the age of 10–13 years^16^. Based on normal eruption time of the maxillary canines at the age of 11 years, 48 (64.9%) patients were treated with surgical exposure before the age of 11 years; this percentage was higher than that in patients aged over 12 years^17^. This shows that, in a clinical situation, orthodontists decide the appropriate timing of intervention for these impacted teeth by evaluating impaction patterns and clinical findings, which are best evaluated by panoramic radiographs rather than expecting spontaneous change of eruption path based on the patients’ age.

CBCT findings associated with impacted maxillary canines were mostly distributed in the following order: cystic-appearing lesion (26.4%), root resorption of adjacent teeth (23.0%), supernumerary teeth (4.6%), and odontoma (4.6%). A cyst is an epithelium lining cavity that contains fluid material. Pathologic analysis of the epithelial lining is mandatory to make a definitive diagnosis^18^. The term ‘cystic-appearing lesion’ is used to distinguish cyst-like lesions on radiographic imaging^19^. Since we were unable to perform pathologic analysis of all of the impacted canines, we chose cystic-appearing lesion as one of the factors that would represent the radiographical characteristics of impacted canines. The ratio of cystic-appearing lesion to resorption of adjacent roots was as high as 49.4% of the total teeth. For orthodontists, presence of pathological signs (cystic-appearing changes and root resorption) is an important factor to determine whether or not to intervene early in the case of impacted canines. In cases of supernumerary teeth and odontoma, orthodontic traction is rarely performed because normal eruption can be expected only by eliminating the cause.

Several studies have confirmed the reliability of panoramic radiographs for diagnosing impacted maxillary canines when compared to that of CBCT and have stated that the sector, encompassing the canines, is an important factor in predicting the normal eruption of the impacted canines^11,12,14^. The impacted maxillary canines treated with surgical exposure and orthodontic traction were mostly distributed in the lateral incisor area (60.9%) and least in the deciduous canine area (8.1%), possibly owing to the primary distribution of impacted canines in the area of maxillary lateral incisors. However, if the impacted canines are close to the deciduous canine area, the chances of intervention by orthodontists are reduced in anticipation of a natural eruption. Conversely, owing to anatomical limitations, the chances of orthodontic traction being performed are reduced if the impacted canines are located close to the maxillary central incisors. Location of maxillary lateral incisors in the middle is suggestive of earlier intervention by orthodontists. The ratio of palatal impaction according to the sector showed a statistically significant correlation. Accordingly, as the impacted maxillary canine was located mesially, the proportion of palatally impacted teeth increased possibly because of the increase in the ratio of palatally impacted canines as they moved to the mesial side. However, it is also possible that orthodontists prefer palatally impacted canines to buccally impacted canines because of the latter’s anatomical limitations, such as contact with adjacent roots when they are on the maxillary central incisor area. A statistically significant correlation was found only in the ratio of resorption of the adjacent roots with respect to the ratio of CBCT findings associated with impacted maxillary canines according to the sectors. In the maxillary central incisor area, half of the 18 impacted canines in sector A showed root resorption in adjacent teeth. This indicates that the proximity of the impacted canine on the mesial side causes root resorption in the adjacent teeth.

In a previous study involving the use of panoramic radiographs to perform various measurements on maxillary canines, the angle of the long axis of the impacted canine and the occlusal plane was 48.7 (± 19.3)°^20^. Similar results were found in the current study at 49.6 (± 17.7)°. Moreover, in previous studies, the distance between the cusp tip of the canine and the occlusal plane was 17.4 mm^13^ or 15.7–20.2 mm^21^. However, a small value of 12.0 mm was found in the current study. This can be due to the magnification rate of the radiographic device used; however, we believe that it can be due to the fact that the samples in this study were impacted canines treated with surgical exposure and orthodontic traction. All the measurements showed statistically significant differences between the sectors, except for 3c-OP. The angle between the long axis of the canine and the occlusal plane decreased when the impacted canine was located mesially. The angle between the long axis of the canine and the midline decreases as the impacted canine is located closer to the deciduous canine area and then increases as it moves away from the deciduous canine area. Based on this aspect, it can be inferred that the dental germ of the impacted canine is mostly located in a certain position near the canine area and only the crown portion of the impacted canine is tilted to the mesiodistal side.

In previous studies involving the treatment period of impacted canines, treatment periods of 5.5 (± 3.4) months for 23 impacted canines^22^ and 8.40 (± 3.26) months for 45 impacted canines in adolescents^23^ were reported. The average duration of traction in the current study was 13.9 (± 8.9) months, which is a longer period than that reported in previous studies. This can be attributed to the fact that the position and traction difficulty of the impacted canines were different from those reported in previous studies. In addition, the definition of treatment completion for impacted canines differs. Since there are many factors involved in the total orthodontic treatment duration, we only limited the traction duration related to impacted canine. We chose a 0.016 × 0.022-inch NiTi wire because when it is engaged, the location, tip, and the rotated state of the impacted canine are aligned roughly. For each sector and the entire tooth, the differences between the traction durations of the buccally or palatally impacted canines were not statistically significant. Hence, only the simple average values were compared. Examination of the entire impacted canines revealed that impacted canines on the palate side (16.2 [± 11.2] months) had a longer traction duration than those on the buccal side (12.9 [± 7.5] months). In sector A, compared to the average of 17.3 (± 5.9) months for palatal impaction, buccally impacted canines took an average of 22.7 (± 17.9) months. This shows that if the maxillary canine is impacted at the position of the maxillary central incisors, anatomical limitations (e.g., contact with adjacent teeth) will result in a longer time and effort taken to perform the traction of the buccally impacted canine.

A statistical analysis of the correlation between the traction duration and the measurements in panoramic radiographs showed that all measurements showed a statistically significant correlation, except for the angle between the long axis of the impacted canine and the occlusal plane. Sajnani and King reported that the distance from the occlusal plane to the cusp tip of the canine is the most important factor for predicting the impaction state^21^. Similar to the results of a previous study, of the four measurement values in this study, the distance from the cusp tip of the canine to the occlusal plane showed the strongest correlation with traction duration (*p* < 0.01). However, this was the only measurement value that did not show statistically significant differences based on sectors. This indicates that when estimating the traction time for treating impacted maxillary canines, the most important consideration is how far the impacted maxillary canine is from the occlusal plane, rather than the angle of the impacted canines.

A limitation of this study is that it was dependent only on diagnoses at a single center when selecting the sample for this study. In addition, we only selected patients treated with surgical exposure and orthodontic traction and did not have additional information when we set up a control group. However, most previous studies focused on predicting the normal eruption of unerupted canines. Thus, there is a lack of research on impacted canines requiring orthodontic intervention, including angle and position on the panoramic radiograph. If a follow-up study is to be conducted in the future, it will be necessary to set up a control group using the diagnosis of a multicenter clinician to focus on the analysis of the characteristics and factors of impacted maxillary canines and to determine whether orthodontic intervention is essential. Furthermore, if we combine the results of this study with recent advancements in artificial intelligence technology, in the near future, simple panoramic images can reveal the expected traction time duration and whether the unerupted canine requires orthodontic intervention.

In conclusion, we found that impacted maxillary canines treated with surgical exposure and orthodontic traction showed increasing possibilities of palatal impaction and resorption of the adjacent root as they were located mesially. Moreover, the distance from the occlusal plane to the impacted maxillary canine showed the strongest positive correlation with traction duration. When establishing treatment plans for patients with impacted maxillary canines, distance from the occlusal plane to the impacted canines, rather than the angle, should be considered in predicting the duration of treatment.

## Methods

This cross-sectional study protocol conformed to the Declaration of Helsinki and was approved by the National Health Insurance Service Ilsan Hospital Institutional Review Board (NHIMC 2022-06-017) and passed the exemption review for informed consent on the use of patients’ radiography and medical records.

### Subjects

This cross-sectional study was conducted based on data of patients aged < 15 years who visited the Department of Orthodontics and National Health Insurance Service Ilsan Hospital between January 2013 and May 2021. Patients were diagnosed as having an “impacted maxillary canine” and underwent surgical exposure and orthodontic traction by two orthodontists with > 20 years of clinical experience. CBCT (CS 9300; Carestream Dental LLC, Atlanta, Georgia, USA) and Panoramic radiograph (CS 8100; Carestream Dental LLC, Atlanta, Georgia, USA) were performed before surgical exposure. The exclusion criteria were as follows: (1) maxillary canines with roots not forming more than two-thirds of the root length, (2) more than three impacted teeth except for the impacted maxillary canines, (3) systemic diseases, and (4) congenital deformities in the craniofacial region, including cleft palate.

A total of 173 patients were selected based on prescriptions for CBCT, surgical exposure, and orthodontic traction; 82 patients were selected based on the inclusion and exclusion criteria. Patients who were referred from local clinics and those who had undergone extraction of impacted canines were excluded. Finally, 74 patients and 87 impacted maxillary canines were included. All 87 teeth were treated using the closed eruption technique, a technique in which a small attachment with a ligature wire is bonded to the impacted canine at the time of exposure and the exposed canine is fully covered by a mucogingival flap as before surgical exposure.

### Cone-beam computed tomography findings associated with impacted maxillary canines

**Figure 1:**
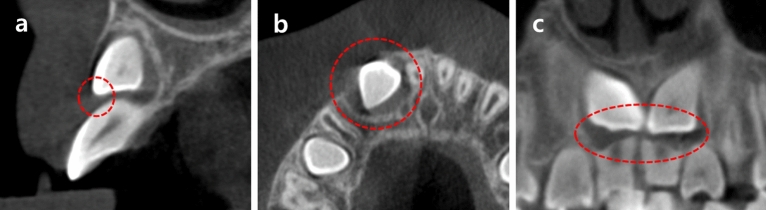
CBCT findings associated with impacted maxillary canines. CBCT findings associated with impacted maxillary canines. (**a**) Bucco-palatal position of impacted maxillary canine (**b**) Cystic-appearing lesion (**c**) Resorption of adjacent root.

CBCT was used to analyze the bucco-palatal position, cystic-appearing lesion, root resorption of adjacent teeth, odontoma, and supernumerary teeth associated with the impacted maxillary canines (Fig. 1). Oral and maxillofacial surgeons confirmed the diagnosis of cystic-appearing lesion radiographically. The bucco-palatal position of the cusp tip of the impacted maxillary canine was determined based on the nearest adjacent teeth.

### Measurements

#### Sector measurement

**Figure 2:**
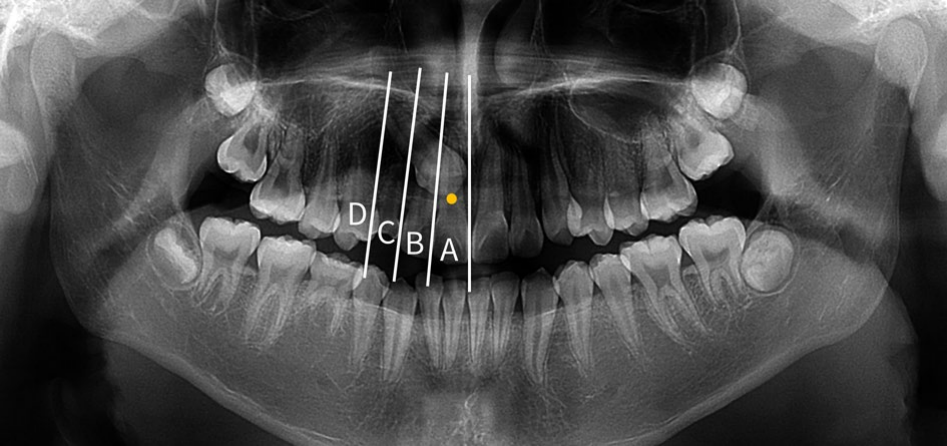
Sector measurement using panoramic radiography. Sector measurement using panoramic radiography. Each sector was divided according to the proximal contact point of adjacent teeth. Impacted maxillary canines were classified by the sector where their cusp tips were located. The figure above shows ‘sector A’.

The sector was divided by drawing a line based on the proximal contact points of the maxillary central incisor, lateral incisor, deciduous canine, and first premolar on panoramic radiographs. The area of the maxillary central incisor, maxillary lateral incisor, deciduous canine, and first premolar was classified as sectors A, B, C, and D, respectively. Impacted canines were classified by sectors based on the location of their cusp tips within each sector (Fig. 2).

#### Angular and linear measurements

**Figure 3:**
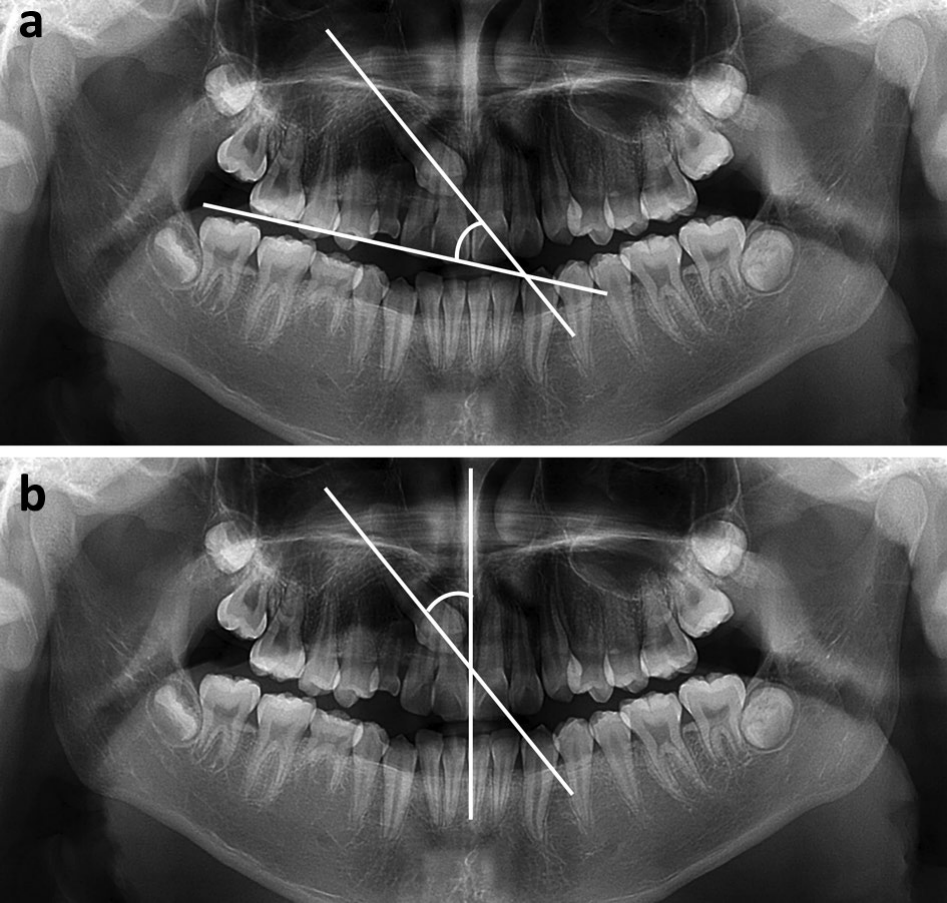
Angular measurements using panoramic radiography. Angular measurements using panoramic radiography. The occlusal plane is defined as the plane which connects the mesiobuccal cusp of the maxillary first molar and the mid-point of incisal edge of the maxillary central incisor. The midline is defined as the line which connects the proximal contact of the maxillary central incisors and anterior nasal spine. (**a**) 3^OP. The angle between the long axis of the canine and the occlusal plane. (**b**) 3^ML. The angle between the long axis of the canine and the midline.

Angles between the long axis of the impacted maxillary canine and the occlusal plane (3^OP) and midline (3^ML) were measured (Fig. 3).

**Figure 4:**
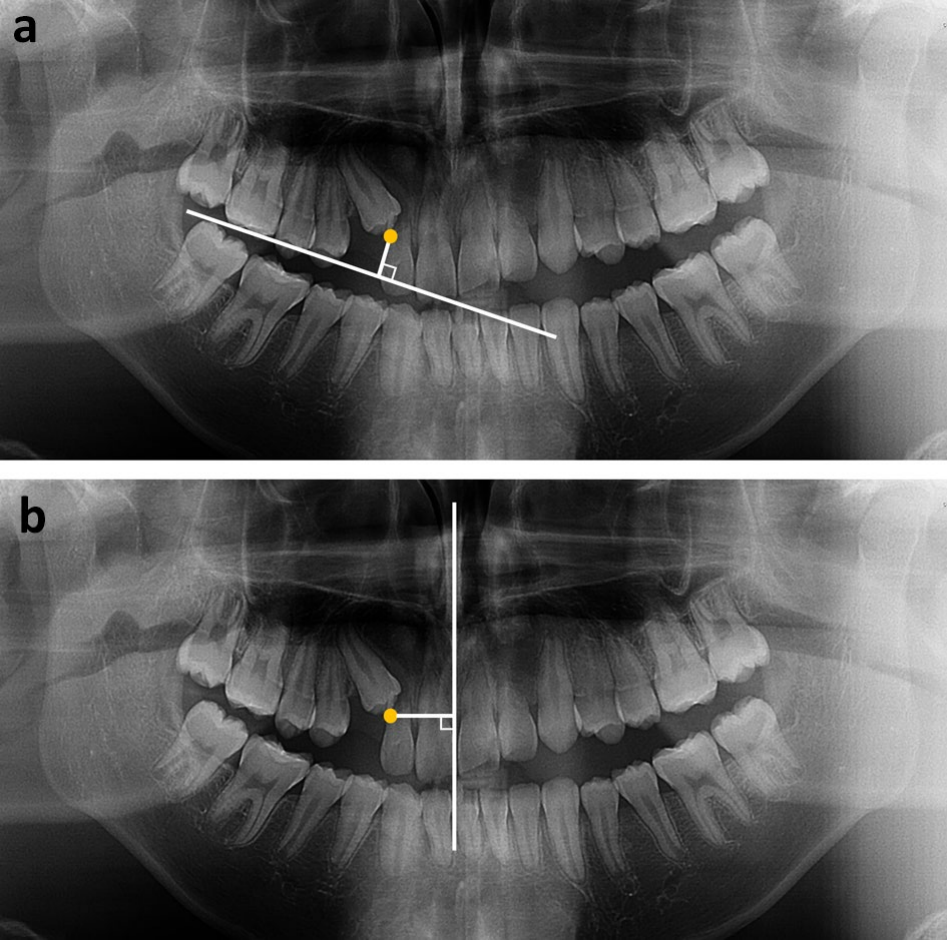
Linear measurements using panoramic radiography. Linear measurements using panoramic radiography. (**a**) 3c-OP. The distance from the cusp tip of the impacted maxillary canine to the occlusal plane. (**b**) 3c-ML. The distance from the cusp tip of the impacted maxillary canine to the midline.

On the panoramic radiograph, the occlusal plane was defined as the plane that connected the mesiobuccal cusp of the maxillary first molar and midpoint of the incisal edge of the maxillary central incisor, and the midline was defined as the line connecting the proximal contact of the maxillary central incisors and anterior nasal spine. Distances from the cusp tip of the impacted maxillary canines to the occlusal plane (3c-OP) and midline (3c-ML) were measured (Fig. 4).

#### Traction duration

Medical records and clinical data were used to investigate the success of orthodontic traction in the management of impacted maxillary canines and the duration spent on orthodontic traction. Traction duration was determined from the time that the orthodontic force was applied to the impacted canine immediately after surgical opening to the time that the impacted canine was aligned enough to engage a 0.016 × 0.022-inch NiTi wire on the maxillary dental arch, rather than the time when the total orthodontic treatment was finished.

### Statistical analyses

Statistical analyses were performed using the Statistical Package for the Social Sciences software (version 23.0; IBM, Armonk, NY, USA). All measurements were performed twice at 2-week intervals by a single researcher. The intra-examiner correlation coefficient showed a high confidence > 0.9 for all the measurements (*p* < 0.001). The Shapiro–Wilk test was performed for normality. A linear-by-linear association test was performed to determine the correlation between the sector and ratio of CBCT findings associated with the impacted maxillary canines. One-way analysis of variance with Tukey’s post-hoc test was performed to determine whether there was a difference between the measurements of 3^OP, 3^ML, 3c-OP, and 3c-ML with respect to each sector. The Mann–Whitney test was performed to determine the difference in traction duration according to the bucco-palatal position of the impacted canines in each sector. The Pearson correlation coefficient was used to determine the correlation between 3^OP, 3^ML, 3c-OP, and 3c-ML and traction duration. P-value less than 0.05 was regarded to be statistically significant.

## Data Availability

The data underlying this article cannot be publicly shared to protect the privacy of the individuals participating in the study. The data will be shared at a reasonable request to the corresponding author.

## References

[1] Hamada Y, Timothius CJC, Shin D, John V (2019). Canine impaction—A review of the prevalence, etiology, diagnosis and treatment. Semin. Orthod..

[2] Becker A, Chaushu S (2015). Etiology of maxillary canine impaction: a review. Am. J. Orthod. Dentofac. Orthop..

[3] Lövgren ML, Dahl O, Uribe P, Ransjö M, Westerlund A (2019). Prevalence of impacted maxillary canines—An epidemiological study in a region with systematically implemented interceptive treatment. Eur. J. Orthod..

[4] Jain S, Debbarma S (2019). Patterns and prevalence of canine anomalies in orthodontic patients. Med. Pharm. Rep..

[5] Arboleda-Ariza N, Schilling J, Arriola-Guillén LE, Ruíz-Mora GA, Rodríguez-Cárdenas YA, Aliaga-Del Castillo A (2018). Maxillary transverse dimensions in subjects with and without impacted canines: A comparative cone-beam computed tomography study. Am. J. Orthod. Dentofacial Orthop..

[6] Potrubacz MI, Chimenti C, Marchione L, Tepedino M (2018). Retrospective evaluation of treatment time and efficiency of a predictable cantilever system for orthodontic extrusion of impacted maxillary canines. Am. J. Orthod. Dentofacial Orthop..

[7] Kim Y, Hyun HK, Jang KT (2017). Morphological relationship analysis of impacted maxillary canines and the adjacent teeth on 3-dimensional reconstructed CT images. Angle Orthod..

[8] Schroder AGD (2018). To what extent are impacted canines associated with root resorption of the adjacent tooth?: A systematic review with meta-analysis. J. Am. Dent. Assoc..

[9] Koç A, Kaya S, Abdulsalam WA (2021). Three-dimensional analysis of impacted maxillary and mandibular canines and evaluation of factors associated with transmigration on cone-beam computed tomography images. J. Oral Maxillofac. Surg..

[10] Brook PH, Shaw WC (1989). The development of an index of orthodontic treatment priority. Eur. J. Orthod..

[11] Ericson S, Kurol J (1986). Radiographic assessment of maxillary canine eruption in children with clinical signs of eruption disturbance. Eur. J. Orthod..

[12] Warford JH, Grandhi RK, Tira DE (2003). Prediction of maxillary canine impaction using sectors and angular measurement. Am. J. Orthod. Dentofacial Orthop..

[13] Margot R (2020). Prediction of maxillary canine impaction based on panoramic radiographs. Clin. Exp. Dent. Res..

[14] Kim S-H (2017). Assessment of the root apex position of impacted maxillary canines on panoramic films. Am. J. Orthod. Dentofacial Orthop..

[15] Ngo CTT, Fishman LS, Rossouw PE, Wang H, Said O (2018). Correlation between panoramic radiography and cone-beam computed tomography in assessing maxillary impacted canines. Angle Orthod..

[16] Ericson S, Kurol J (1988). Early treatment of palatally erupting maxillary canines by extraction of the primary canines. Eur. J. Orthod..

[17] Kang TS, Choi BJ, Son HK, Choi HJ, Kwon HG (2005). Timing and sequence of eruption of permanent teeth in a sample of children from Yonsei dental hospital. J. Kor. Acad. Pediatr. Dent..

[18] Devenney-Cakir B, Subramaniam RM, Reddy SM, Imsande H, Gohel A, Sakai O (2011). Cystic and cystic-appearing lesions of the mandible. AJR. Am. J. Roentgenol..

[19] Oda M, Staziaki PV, Qureshi MM, Andreu-Arasa VC, Li B, Takumi K, Sakai O (2019). Using CT texture analysis to differentiate cystic and cystic-appearing odontogenic lesions. Eur. J. Radiol..

[20] Kim HJ, Park HS, Kwon OW (2008). Evaluation of potency of panoramic radiography for estimating the position of maxillary impacted canines using 3D CT. Kor. J. Orthod..

[21] Sajnani AK, King NM (2012). Early prediction of maxillary canine impaction from panoramic radiographs. Am. J. Orthod. Dentofacial Orthop..

[22] Becker A, Chaushu S (2003). Success rate and duration of orthodontic treatment for adult patients with palatally impacted maxillary canines. Am. J. Orthod. Dentofacial Orthop..

[23] Arriola-Guillén LE, Aliaga-Del Castillo A, Ruíz-Mora GA, Rodríguez-Cárdenas YA, Dias–Da Silveira HL (2019). Influence of maxillary canine impaction characteristics and factors associated with orthodontic treatment on the duration of active orthodontic traction. Am. J. Orthod. Dentofacial Orthop..

